# The social determinants of health facility delivery in Ghana

**DOI:** 10.1186/s12978-019-0753-2

**Published:** 2019-07-10

**Authors:** Emmanuel Dankwah, Wu Zeng, Cindy Feng, Shelley Kirychuk, Marwa Farag

**Affiliations:** 1 School of Public Health, University of Saskatchewan, 104 Clinic Place, Saskatoon, SK S7N 2Z4 Canada; 2 College of Medicine, Canadian Centre for Health and Safety in Agriculture (CCHSA), University of Saskatchewan, 104 Clinic Place, Saskatoon, SK S7N 2Z4 Canada; 3 The Heller School for Social Policy and Management, Brandeis University, 415 South St, Waltham, MA 02453 USA; 4 School of Public Administration and Development Economics, Doha Institute for Graduate Studies, Al Tarfa street, Zone 70, Doha, Qatar

**Keywords:** Health facility, Delivery, Reproductive aged women,, Social determinants, Birth

## Abstract

**Background:**

Many women still deliver outside a health facility in Ghana, often under unhygienic conditions and without skilled birth attendants. This study aims to examine the social determinants influencing the use of health facility delivery among reproductive-aged women in Ghana.

**Methods:**

Nationally representative data from the 2014 Ghana Demographic and Health Survey was used to fit univariable and multivariable logistic regression models to estimate the influence of the social determinants on health facility delivery. Andresen’s health care utilization model was used as the conceptual framework guiding this study..

**Results:**

Only 72% of deliveries take place at a health facility in Ghana. The results of the adjusted model indicate that place of residence, financial status, education, religion, parity and perceived need were significantly associated with health facility delivery. First, urban women had a higher likelihood of health facility delivery than rural women (Adjusted Odds ratio [AOR] =2.21; 95% Confidence interval [CI] = 1.53–3.19). Second, middle-class and rich women were 1.57 (95%CI = 1.18–2.08) times and 6.91 (95%CI = 4.12–11.59) times, respectively more likely to deliver at health facility compared to the poor. Third, women with either at least secondary education (AOR = 2.04; 95%CI = 1.57–2.64) or primary education (AOR = 1.39, 95%CI = 1.02–1.92) were more likely to deliver at health facility than women with no education. In terms of parity, first time mothers were 1.58 (95% CI = 1.18–2.12) times more likely to deliver at health facility than those who had given birth three or more times before. Finally, regarding perceived need, women who were aware of pregnancy complications were 1.32 (95%CI = 1.02–1.70) times more likely to use health facility delivery than those who were not informed about pregnancy complications.

**Conclusions:**

First, in spite of Ghana’s free maternal health services policy, poorer women were much less likely to have a health facility delivery, which points to the need to understand the indirect costs and other financial barriers preventing women from delivering at a health facility. Second, many of the identified variables influence the demand and not just the supply for health care services, and highlight the importance of the social determinants of health and investments in interventions that extend beyond improving physical access.

## Plain English summary

Delivery outside a health facility in a developing country context often occurs under unhygienic conditions and in the absence of professionally trained skilled birth attendants. Hence, delivery outside a health facility is linked to increased risk of delivery complications including obstructed labour, retained placenta, postpartum haemorrhage among others. Further, deliveries at outside health facility have contributed to maternal and neonatal mortality. Regrettably, considerable proportions of Ghanaian women still deliver outside a health facility even though health facility delivery has been shown to reduce complications associated with childbirth. This research examined factors potentially influencing women’s use of health facility delivery to generate evidence that could help direct public policy towards more effective interventions. In spite of Ghana’s free maternal health services policy, the findings indicate that about 72% of Ghanaian women in the childbearing age delivered at health facility. The results of the study revealed that, as expected, women who are poorer, with lower education, who reside in rural areas, who have previously given birth, and were not informed about pregnancy complications, had increased risk of delivering outside a health facility. These findings indicate that factors other than physical access matter a lot in influencing a woman’s decision to deliver at a health facility and hence targeted interventions that focus on women who are at higher risk of delivering outside a health facility are needed.

## Introduction

Every year, there are a over 200 million conceptions [1]. However, about 40% of these conceptions result in pregnancy-related problems among women around the world [1]. Childbirths in health facilities have been recognized as one of the best strategies to avoid maternal mortalities and morbidities and to improve the health of newborns [2–4]. Despite this recognition, a significant percentage of childbirths occur outside health facilities in low-income countries [3, 5]. One of the tragic consequences of this underutilization is the 830 maternal deaths that occur each day because of pregnancy and labour-related complications [6]. If this consequence is not tragic enough, delivery outside facility also has ramifications for infants. Delivery outside of health facilities contributed to annual neonatal mortalities of 3 million [7] and 2.65 million stillbirths globally in 2008 [8]. Further, home delivery is linked with increased risk of third stage delivery issues including retained placenta, postpartum haemorrhage [9].

Ghana has a population of about 28 million and a population density of 124. Females account for about 51 % of the population with an estimated total fertility rate of 4 [6]. According to the Ghana Demographic and Health Survey report [10], inhabitants reside almost equally in urban and rural areas. In Ghana, health facilities administer health care through maternity homes, Community-based Health Planning and Services (CHPS) health post, public, private and mission hospitals. The distribution of health care facilities is skewed in favour of the urban areas.

Maternal deaths continues to be unjustifiably high, though the number of deaths has almost been halved from 634 per 100,000 live births in 1990 to 319 per 100,000 live births in 2015 [6]. This reduction is thought to be the result of the introduction of free maternal care policy, antenatal care (ANC) services and increased institutional deliveries [11–13]. However, even though most pregnant Ghanaian women seem to use ANC services, a large percentage of deliveries still take place outside health facility. This trend is supported by data collected in the 2008 Ghana Demographic and Health Survey (GDHS): 95% of pregnant women reported utilizing ANC services from skilled personnel, including medical doctors, midwives, and nurses. Nonetheless, only 59% delivered at health facility in the presence of health professional in 2008 [14]. The Government of Ghana has introduced initiatives such as free maternal health care services, Community-based Health Planning and Services (CHPS), and improved antenatal care and education in an effort to improve access to health facility delivery; these initiatives have been successful in increasing the use of health facility deliver but it remains inequitably distributed [15, 16]., According to a 2011 survey, 37% of childbirths occurred at health facility in Northern Ghana, and 52.7% of deliveries occurred at health facility in rural Ghana [17]. Both of these percentages were well below the national health facility delivery rate of 67.4% in 2011 [17]. Thus, more efforts are needed to ensure equitable access to this potentially life-saving surgery.

Understanding the determinants of health facility delivery is important for targeting policies and interventions. A body of literature has found that socio-economic and demographic dynamics affect women’s choice of birthplace [18–27], however, inconsistency about how these factors influence women’s decisions remains a major concern. In Ghana, some studies have investigated the effect of socio-demographic characteristics on health facility delivery [18, 23, 28, 29]. Notwithstanding, these studies were not exhaustive, and highlight the need for further research. For example, none of the studies considered the effect of ‘need’ on the use of health facility as a place for delivery. Also, previous studies did not use nationwide data making it inaccurate to generalize findings to the entire population. The present study aims to examine social determinants influencing the use of health facility delivery among Ghanaian women of reproductive age.

## Methods

### Study data

The 2014 Ghana Demographic and Health Survey (GDHS) dataset was used in this study after permission from the MEASURE DHS. GDHS was carried out by the Ghana Statistical Service (GSS), ICF international and Ghana Health Service. The survey used two-stage systematic sampling to select participants from households nested in clusters (enumeration areas) across the all the 10 regions of Ghana. The survey interviewed 9396 women aged 15–49 years with a response rate of 97% [10]. The GDHS collected information on socio-demographic characteristics and reproductive health issues. Detailed information on the sampling techniques and the questionnaires have been reported elsewhere [10].

### Conceptual framework for health facility delivery

**Fig. 1:**
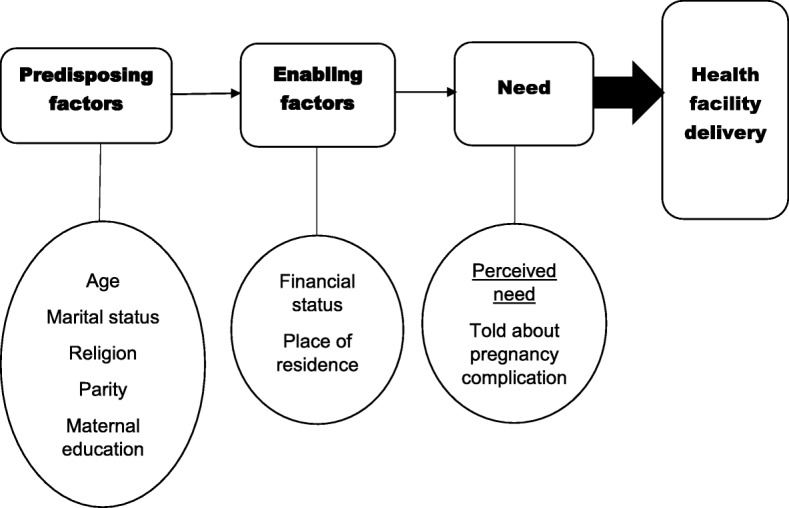
Theoretical framework adapted from Andersen’s Health utilization model

This research adopted and modified the Andersen’s healthcare utilization model to study obstetric services use (place of delivery). Andersen’s model considers three types of factors as drivers of health services’ use: predisposing, enabling and need factors [30–32].. First, predisposing factors refer to characteristics that exert influence prior to the occurrence of the given health behaviour, by encouraging or inhibiting the uptake of health facility delivery in this case. Predisposing characteristics include all characteristics that might condition individual’s perceptions of need and use of health facility delivery. These predisposing factors can take the form of demographic factors (age), reproductive history (parity), cultural beliefs (religion), civil status (marital status), and social structure/factors (education) among other factors [31, 32]. Second, enabling factors are related to the resources that facilitate or impede the utilization of health services which include financial status, resources in the community and other factors [31, 32]. Third, Andersen’s model proposes that “**Need**” for care is important for influencing behavior [31]. Andersen’s model, in addition to an extensive review of empirical literature [18, 23, 25, 26], was used as guide to select potential factors associated with health facility delivery. The explanatory predictors considered in the study were grouped into predisposing factors (age, marital status, religion, parity, maternal education), enabling factors (financial status and place of residence) and perceived need as shown in Fig. 1. Since this dataset does not include a variable representing actual medical need for health facility delivery, the variable ‘told about pregnancy complications’ was used as a proxy for perceived need for health facility delivery; the choice of this variable as a proxy for need is based on another study that used a similar variable [33].

### Study variables

The study sample included 4293 women of reproductive-age (15 to 49 years) who responded to the question asking about place of delivery. Birth outside health facility, hereinafter was referred to as home delivery. The outcome variables were coded as follows: health facility delivery = ‘1’ and, home delivery = ‘0’. Eight explanatory variables were used: (1) maternal age, (2) financial status, (3) education, (4) place of residence, (5) religion, (6) marital status, and (7) parity, and (8) having been told about pregnancy complications.

Maternal age violated the linearity assumption with a significant quadratic term (*p*-value = 0.001) and hence was categorized. As shown in Table 1, explanatory variables were grouped as follows: (1) Maternal age (15–24 years, 25–34 years, 35–49 years); (2) The Financial status variable was created from the Wealth index variable that was available in GDHS dataset. The GDHS generated the Wealth index based on information on household assets using principal component analysis (PCA). The Wealth index variable was comprised of 5 categories namely poorest, poorer, middle, richer and richest. The Financial status variable used in this study is comprised of 3 categories: poor (poorer, poorest), middle, and rich (richer, richest) as reported in a similar studies. [25, 34]; (3) Education was classified into no education, primary (grade 1–6), at least secondary (above grade 6); (4) place of residence (urban, rural); (5) religion (Christian, Islam, Traditional and Others); (6) marital status (married, unmarried); (7) parity (1 birth, 2 births, 3 births or more); (8) told about pregnancy complications (yes, no).

**Table 1:** Study variable description

Variable name	Description	Level of measurement
Place of delivery	Respondents place of delivery	0 = Home delivery
		1 = Health facility
Age	Maternal age (years)	1 = 15 to 24
		2 = 25 to 34
		3 = 35 to 49
Financial status	Financial status of the household	1 = Poor
		2 = Middle
		3 = Rich
Marital status	Current marital status	0 = Unmarried
		1 = Married
Residence	Place of residence	1 = Urban
		2 = Rural
Education	Highest educational level	1 = No Education
		2 = Primary
		3 = at least Secondary
Religion	Religious affiliation of the women	1 = Christians
		2 = Islam
		3 = Traditional/other
Parity	Number of Births	1 = 1 birth
		2 = 2 births
		3 = 3 or more births
Pregnancy complications	Told about pregnancy complications	No = 0
		Yes = 1

### Statistical analyses

The present study used sampling weights provided by GDHS. The weighting factor from the survey was used to address sampling error and non-response to ensure validity of the findings. All analyses were conducted in SAS version 9.4 (SAS Institute, Cary, NC, USA). Proportions and frequencies were tabulated for each of the categorical independent variables. The effect of the risk factors on health facility delivery were fitted using a logit model stated as:

$$ p\left( Yi=1\right)= pi $$

$$ \mathrm{logit}\ \left(\mathrm{pi}\right)={\upbeta}_0+{\upbeta}_1{\mathrm{X}}_1+{\upbeta}_2{\mathrm{X}}_2\dots +{\upbeta}_{\mathrm{k}}{\mathrm{X}}_{\mathrm{k}\mathrm{i}} $$

where pi is the probability of health facility delivery among the i th women; β_0_ is the intercept; β_k_ is the regression parameters; Xi is the independent variables; Yi is the outcome of interest.

This study employed Taylor series linearization method, which is a variance estimation procedure used by PROC SURVEYLOGISTIC in SAS, to adjust for clustering effect. Univariable logistic regression models were used to screen independent variables for further analysis. In the unadjusted model, risk factors with a liberal of *p*-value ≤0.25 were selected for inclusion in the multivariable logistic regression model based on the Hosmer and Lemeshow publication [35]. This lenient p-value cut-off was used to prevent missing important factors whose effect could be suppressed or concealed by confounding effect. Multicollinearity among select independent variables for the adjusted model was checked, variance inflation factor (VIF) greater than 2.5 was considered collinear. A multivariate logistic regression model was fitted to examine the link between health facility delivery and explanatory factors. The manual backward elimination technique was employed for model building in this research [36]. First, all factors that were selected for the adjusted model were included. Factors with the highest *p*-values and widest 95% confidence intervals were selected for elimination from the model one at a time until all the predictors in the model were significant at *p*-value ≤0.05.

The odds ratios and corresponding 95% confidence intervals were computed for all the significant variables in the adjusted model.. Two-way interactions among significant factors in the adjusted model were tested. The model with the smallest Akaike’s Information Criterion (AIC) was selected as a parsimonious model.. Further, area under the Receiver Operating Characteristic (ROC) was used for model diagnostics to assess the discriminatory power of the model on the study outcome.

## Results

### Descriptive results

Four thousand two hundred ninety three women between the ages of 15 and 49 responded to the place of delivery question in the GDHS 2014 (Table 2). The study population was reasonably young with an average age of 29.7 years (SD = 9.71). About 72% of women reported health facility delivery, while 28% used home delivery.

**Table 2:** Distribution of health facility delivery by social determinants (*N* = 4293)

Predictors	N (%)	Health facility delivery *N* = 3107(%)	*P*-value (Chi-Sq)
Age			
15–24	922 (21.4)	681 (73.9)	0.1
25–34	2026 (47.6)	1480 (73.0)	
35–49	1345 (31.0)	946 (70.3)	
Place of Residence			
Urban	1777 (46.2)	1595 (89.8)	< 0.0001*
Rural	2516 (53.8)	1512 (60.0)	
Education			
No Education	1419 (26.1)	766 (54.0)	< 0.0001*
Primary	869 (19.6)	602 (69.3)	
Secondary and above	2005 (54.3)	1739 (86.7)	
Financial status			
Poor	2241 (41.3)	1287 (57.4)	< 0.0001*
Middle	812 (20.0)	634 (78.0)	
Rich	1240 (38.7)	1186 (95.6)	
Marital Status			
Married	2801 (61.7)	2035 (72.7)	0.6
Unmarried	1492 (38.3)	1072 (71.8)	
Religion			
Christian	3047 (71.0)	2321 (76.2)	< 0.0001*
Muslim	922 (21.4)	671 (72.8)	
Traditional/other	324 (7.6)	115 (35.5)	
Parity of the women			
1 birth	934 (22.8)	790 (84.6)	< 0.0001*
2 births	839 (20.3)	657 (78.3)	
3 or more births	2520 (56.9)	1660 (65.9)	
Told about pregnancy complications^±^			
Yes	3423 (82.5)	2579 (75.3)	< 0.0001*
No	724 (17.5)	496 (68.5)	

*N* number of observations; %, percent; ± *N* = 4147, due to missing observations; *significant at *p*-value of 0.005; 2014 GDHS data

#### Age

Almost half (47.6%) of the women fell between the ages of 25–34 years. About one-third (31%) of the women’s ages ranged between 35 and 49 years, while roughly 1 in 5 (21%) were between 15 and 24 years. Across these age groups, similar trends were observed. About three-quarters (73.9%) of the women aged 15–24 years delivered at health facility. Likewise, 73% and 70.3% reported having health facility delivery among 25–34 and 35–49-year-olds, respectively (Table 2).

#### Residence

More than half (53.8%) of the women lived in rural areas. Among these rural dwellers, about two-thirds (60%) used health facility for delivery whereas approximately 9 in 10 (89.8%) urban dwellers delivered at health facility (Table 2).

#### Education

Over a half (54.3%) of the women had attained at least secondary education. About a quarter (26.1%) of the women had no formal education, and nearly one-fifth (19.6%) had attained primary education. Regarding health facility delivery, more than half (54%) of the women with no education reported having had delivery at a health facility. For women with primary and at least secondary education, health facility delivery was 69.3 and 86.7% respectively (Table 2).

#### Financial status

The “poor” category included about 41.3% of the population while the “middle” income group included about 20%, and more than one-third (38.7%) of the women were in the “rich” group. Regarding delivery place, roughly three-fifths (57.4%) of the poor women reported having had health facility delivery. Among middle-class and rich women, 78 and 95.6% births respectively occurred at health facility (Table 2).

#### Marital status

The majority (61.7%) of the women were married. Out of married women, health facility deliveries accounted for 72.7%. Similarly, 71.8% of unmarried women reported having health facility delivery (Table 2).

#### Religion

Most of the women (71.0%) were Christians. The second largest religious group was Muslim (21.4%), followed by traditional and other beliefs (7.6%). Health facility delivery trends were alike among Christian and Muslim women, but there was a marked difference among women with traditional and other beliefs. The study found that about three-quarters of Christians (76.2%) and Muslims (72.8%) reported having had health facility delivery. Also, a much smaller percentage (35.5%) of women with traditional and other beliefs had delivery at health facility (Table 2).

#### Parity

More than half (56.9%) of the women had given birth at least 3 times whilst 22.8% of the women had given birth once, and 20.3% had given birth twice. Among first time mothers, 84.6% delivered at health facility. Also, 78.3% of women who had given birth twice used health facility for delivery. Lastly, among mothers with three or more children, 65.9% of deliveries took place at health facility (Table 2).

#### Perceived need

Women who were informed about pregnancy complications had health facility delivery higher than their counterparts who were not aware of pregnancy complication (75.3% vs. 68.5%).

### Univariable analysis results

The associations between select social determinants and health facility delivery were tested (Table 3). Unadjusted analyses revealed the following associations:

**Table 3:** Unadjusted Odds ratio and 95% confidence interval of having health facility delivery by socio-demographic risk factors in the univariable logistic regression analyses

Predictors	Unadjusted Odds Ratio (95% Confidence Interval)	*P*-value
Age (Ref: 15-24)		
25–34	1.09 (0.91, 1.30)	0.6
35–49	1.03 (0.81, 1.31)	
Residence (Ref: Rural)		
Urban	6.71 (4.76, 9.44)	< 0.0001
Highest Education Level (Ref: No education)		
Primary	1.89 (1.40, 2.55)	< 0.0001
Secondary and above	5.92 (4.43, 7.90)	
Financial status (Ref: Poor)		
Middle	2.69 (2.03, 3.59)	< 0.0001
Rich	18.55 (12.54, 27.44)	
Religion (Ref: Traditional/other)		
Muslim	4.20 (2.43, 7.26)	< 0.0001
Christian	5.85 (3.79, 9.0)	
Marital status (Ref: Unmarried)		
Married	1.05 (0.85, 1.28)	0.7
Parity of the women (Ref: 3 or more births)		
1 birth	2.77 (2.19, 3.50)	< 0.0001
2 births	1.73 (1.35, 2.21)	
Told about pregnancy complications (Ref: No)		
Yes	1.65 (1.29, 2.11)	< 0.0001

Ref, reference; %, percent; 2014 GDHS data

#### Age

The study found a weak association between the age of a woman and health facility delivery. The univariable analysis produced point estimates a little over one for women aged 25–34 years (Unadjusted OR = 1.09; 95%CI = 0.91–1.30) and those aged 35–49 years (Unadjusted OR = 1.03; 95%CI = 0.81–1.31) relative to women aged 15–24 years. Additionally, the overall *p*-value (0.6) was greater than 0.25, and hence was not selected for the multivariable logistic regression model (Table 3).

#### Residence

Women living in urban areas were 6.71 (95%CI = 4.76–9.44) times more likely to deliver at health facility than rural residents (Table 3).

#### Education

Compared to women with no education, secondary or higher educated women 5.92 (95%CI = 4.43–7.90) times more likely to deliver at health facility. Women with primary education were 1.89 (95%CI = 1.40–2.55) times more likely to report having health facility delivery than women with no education (Table 3).

#### Financial status

The odds of having health facility delivery among rich women were about 18.55 (95%CI = 12.54–27.44) times when compared to the poor. Moreover, middle class women were 2.69 (95%CI = 2.03–3.59) times more likely to deliver at health facility than poor women (Table 3).

#### Religion

Muslims and Christians were 4.20 (95%CI = 2.43–7.26) times and 5.85 (95%CI = 3.79–9.0) times respectively, more likely to deliver at health facility (Table 3).

#### Marital status

The relationship between marital status and health facility delivery in this study was not significant (unadjusted OR = 1.05; 95%CI = 0.85–1.28). Further, *p*-value (0.7) higher than 0.25 was identified. In view of that, marital status was not considered for further analysis in the adjusted model (Table 3).

#### Parity

The higher the number of times a woman give birth, the less likely to resort to health facility delivery in subsequent births. The results revealed that women who had given birth twice were 1.73 (95%CI = 1.35–2.21) times more likely to deliver at health facility than mothers with three or more births. First-time mothers were 2.77 (95%CI = 2.19–3.50) times more likely to have health facility delivery than women who had given birth three or more times.

#### Perceived need

Women who were aware of pregnancy complications had a higher likelihood of using health facility for delivery relative to women who were not informed about pregnancy complications (unadjusted OR = 1.65; 95%CI = 1.29–2.11).

### Multivariable analysis results

Only the maternal age and marital status variables were not considered in the adjusted model. In this study, the significant factors associated with health facility delivery in the multivariable logistic regression analysis are presented in Table 4.

**Table 4:** Adjusted Odds ratio and 95% Confidence interval of having health facility delivery by socio-demographic risk factors in the multivariable logistic regression analyses

Predictors	Adjusted Odds Ratio (95% Confidence Interval)	*P*-value
Residence (Ref: Rural)		
Urban	2.21 (1.53, 3.19)	< 0.0001*
Highest Education Level (Ref: No education)		
Primary	1.39 (1.02, 1.92)	0.04*
Secondary and above	2.04 (1.57, 2.64)	< 0.0001*
Financial status (Ref: Poor)		
Middle	1.57 (1.18, 2.08)	0.002*
Rich	6.91 (4.12, 11.59)	< 0.0001*
Religion (Ref: Traditional/Other religion)		
Muslim	2.75 (1.61, 4.69)	< 0.0001*
Christian	2.53 (1.67, 3.84)	< 0.0001*
Parity of the women (Ref: 3 or more births)		
1 birth	1.58 (1.18, 2.12)	0.002 *
2 births	1.07 (0.82, 1.39)	0.6
Told about pregnancy complications (Ref: No)		
Yes	1.32 (1.02, 1.70)	0.03*

Ref, reference; %, percent; *significant at *p*-value of 0.05; 2014 GDHS data

#### Residence

Women living in urban areas were 2.21 (95%CI = 1.53–3.19) times more likely to use health facility for delivery than their rural counterparts.

#### Education

Women who attained at least secondary education were 2.04 (95%CI = 1.57–2.64) times more likely to deliver at health facility than uneducated women. Likewise, women who had a primary education were 1.39 (95% CI = 1.02–1.92) times more likely to have health facility delivery relative to those without education.

#### Financial status

The odds of having health facility delivery were 6.91 (95%CI = 4.12–11.59) times higher among the rich than poor women. Compared to poor women, middle-level women were 1.57 (95%CI = 1.18–2.08) times more likely to deliver at health facility.

#### Religion

The likelihood of health facility delivery among Christians was 2.53 (95%CI = 1.67–3.84) times higher than traditional and other believers. Likewise, Muslims were 2.75 (95%CI = 1.61–4.69) times more likely to deliver at health facility than traditional and other believers.

#### Parity

Odds of having health facility delivery among women who had given birth once were 1.58 (95%CI = 1.18–2.12) times higher than mothers with 3 or more births. Conversely, no significant difference was detected between women who had given birth twice and those with three or more childbirth experience (Adjusted Odds ratio = 1.07; 95%CI = 0.82–1.39)).

#### Perceived need

The analysis revealed that women who were aware of pregnancy complications were **1.**32 (95%CI: 1.02–1.70) times more likely to deliver at health facility when compared with women who were not informed about pregnancy-related issues.

## Discussion

Home delivery especially without skilled supervision is a major concern not only in Ghana but also in other developing countries including Kenya [27] and India [26]. Even after the introduction of CHPS and the implementation of a health policy that granted free access to maternal health care, a significant proportion of Ghanaian women still deliver at home. Our study revealed that about 72% of childbirths in Ghana occur at health facility. Though, this percentage is an improvement from the 61.9% health facility delivery rate reported by Boah et al. [28], yet it is unacceptably low if the goal is to achieve universal coverage in terms of health facility delivery.

In Ghana, there are a number of factors that ultimately influence women’s decision about the place of their birth. These factors are dynamic, and reflect the complex nature of childbirth, women’s autonomy, and familial relationships. In our study, women’s age and marital status were not significantly associated with health facility delivery. These results are supported by some of the literature [19, 25]. However, other literature suggests that these factors may have predictive value [22, 24, 25, 34]. Recognizing that there is a discrepancy is important. Such a discrepancy suggests that more needs to be done to effectively contextualize women’s decision-making, and the factors that will and will not be predictive given that context.

Based on the results of our study, factors that may be important for women’s decision-making about the place of their delivery include place of residence, education, financial status, religion, parity and perceived need. Recognition of a host of factors that have the potential to be predictive of women’s decision-making reflects the need to take a public health approach that emphasizes the social determinants of health when examining health facility deliveries. A social determinants approach recognizes that health extends far beyond the medical model. Using a social determinants’ lens to improve health facility deliveries has the potential to transform Ghanaian women’s birthing experiences, health outcomes, and their children’s wellbeing. However, the relevance of each of our study’s factors must be assessed in light of the literature.

### Residence

Place of residence tends to influence the choice women make about place of delivery [37]. In our study, only about half of births among women living in rural areas occurred at a health facility, and this is consistent with statistics reported by Ghana Health Service [38]. Women living in urban areas were more likely to deliver at health facility than rural dwellers in the multivariate regression model controlling for other important factors. These findings are consistent with results reported in other African countries, including Ethiopia [39], Nigeria [40], and Kenya [24]. The disparity between rural and urban women may be a consequence of the physical location of health facilities. That is, more maternal health facilities are located in urban areas, meaning that those facilities are more accessible to the women that live there. Urban women do not face the same barriers to physical access that rural women do; poor roads and the remoteness of some communities mean that health facility delivery may be not be a viable option for some rural women. Further, the proximity of health facilities in urban areas means there is a more concrete network between various health services, and urban women may be able to receive referrals and make use of multidisciplinary teams to a greater extent than rural women. Given the strong association between place of residence and home delivery, efforts directed at improving rural health services in Ghana may be warranted.

### Education

A woman’s choice regarding where she delivers her child and her education level are closely linked [27]. Upon holding everything else constant, as the level of education increased, the likelihood of health facility delivery increased. This finding is consistent with other studies [19, 23, 34, 41, 42]. This trend is thought to be a function of the improved health literacy among educated women. That is, educated women are better able to understand and become informed about health care issues. As a result, their health care decisions reflect this awareness [43]. This finding indicates that creating public health programming that targets women with lower levels of education may be an effective way to increase the number of health facility deliveries.

### Financial status

In our study, the poorer the woman, the less likely it is that she delivers at health facility. This trend is mirrored in studies conducted in Kenya [27, 43], Ghana [25], and Nigeria [42]. Given that Ghana’s maternal health care is free, our results suggest that, aside from the cost of health services, other economic factors influence women’s decision-making when it comes to choice of delivery place. This nuanced interpretation draws a crucial distinction between the Ghanaian context and other jurisdictions, and must be explored. This interpretation is affirmed in some literature, where a women’s inability to purchase maternal health services was not the sole reason for opting to deliver outside of a health facility [24]. Other contributing factors may include: the cost of transportation, time spent traveling, and miscellaneous fees associated with receiving care in health facilities [44, 45]. This broad interpretation of the costs associated with health facility deliveries is crucial for gaining a deeper understanding of how financial status influences women’s decisions to resort to home delivery.

### Religion

Women who were Christians or Muslims were more likely to deliver at health facility than those who had traditional and other beliefs. This result is supported by other research conducted in Ghana [18], but contrary to the results from a research study conducted in Uganda [19]. Given the differences in socio-demographics between Uganda and Ghana, the discrepancy may be a function of the different religious make-ups. The reason why women with traditional and other beliefs are less likely to deliver at health facility may be because of their opposition to modern health services. These women may perceive pregnancy and labour as natural occurrences that should be free of medical intervention, except in the case of an emergency [5, 46]. Considering that religion is highly personal, public health interventions aimed at connecting these women with maternal health services must be developed with the utmost cultural competence to help discourage home deliveries and its associated consequences.

### Parity

The number of times a woman had given birth was strongly associated with health facility delivery. As birthing times increased, the likelihood of a home delivery decreased. In a similar study conducted in rural south Ghana, first-time mothers were more likely to have health facility delivery than women who had previously given birth more than twice [23]. This direct association is consistent with findings from literature [24, 27, 41]. One possible explanation for this finding could be that if a woman received poor health services during her previous deliveries, she may be less likely to access those services again [24, 47, 48]. Apart from these negative experiences, self-confidence from previous labours [19], lower complications from previous pregnancy, and the notion that home delivery is a sign of bravery [5], tend to aggravate the likelihood of delivering at home. Unpacking this trend is important because if the quality of maternal health services is detracting women from using them, then improvements must be made. Further investigation is needed to examine the association between quality of maternal health care and health facility delivery among multiparous. Given the data source, this was not possible in the current study. However, such an investigation would provide important insights about the quality of maternal health care services, and their ability to adequately meet expectant mothers’ needs. Eventually, it would assist in improving health facility deliveries among multiparous women.

### Perceived need

The study identified that health facility delivery was significantly associated with perceived need of the mothers but the association was not strong. Perceived need i.e. knowledge about pregnancy complications was associated with health facility delivery in this study. The study result is consistent with some earlier studies in the African sub-region [28, 33, 49] that found greater use of health facility delivery among mothers who were informed about pregnancy and delivery issues. This result exposes the quality of health information mothers received during antenatal care (ANC) since the rate of ANC uptake have been reported to be very high in Ghana by other studies [10, 28]. This finding points to the need to give increased attention to health education about potential delivery associated complications as part of ANC in Ghana.

#### Study strength and limitations

This study used the most recent nationally representative Demographic and Health survey data to examine the social determinants of health facility delivery use. The results from the present study contributes to the body literature on the social determinants of health and highlights the need for targeted maternal health programming especially in developing economies. However, this research was characterized with some limitations which should be recognised in the interpretation of the findings. A major limitation in this study is that data pertaining to the socio-demographic factors was collected during the survey period, and not at the time of delivery. Also, as result of lack of data on actual medical need, the variable “told about pregnancy complications” was used as a proxy for perceived need and hence conclusions based on this variable should be drawn with caution.

## Conclusion

In conclusion, this study adds to the body of international literature on the social determinants of health as it finds that there is a host of factors that influences Ghanaian women’s decision about health facility delivery. Many of these factors are demand side factors and hence improving physical access alone is unlikely to create the important changes needed to increase health facility delivery among Ghanaian women and improve equitable access to it. In addition, in spite of Ghana’s free maternal health care policy, poorer women were much less likely to have a health facility delivery, which raises the issue of other indirect financial barriers to access and the importance of tackling these barriers. Taken together, there is a need for effective maternal health programming to target poorer, less educated women who reside in rural areas, and who have previously given birth to increase health facilities deliveries in Ghana and hence improve maternal health outcomes.

## Data Availability

The datasets analyzed during the current study are available in the MEASURE DHS repository, https://www.dhsprogram.com/data/dataset_admin/login_main.cfm. ^[GHIR72DT]^

## Abbreviations

AIC: Akaike’s Information Criterion
ANC: Antenatal Care
AOR: Adjusted Odds Ratio
AUC: Area under the curve
CHPS: Community-based Health Planning and Services
CI: Confidence Interval
GDHS: Ghana Demographic and Health Survey
GSS: Ghana Statistical Service
OR: Odds Ratio
PCA: Principal Component Analysis
ROC: Receiver operating characteristics
SD: Standard deviation
VIF: Variance Inflation Factor
WHO: World Health Organization
